# A Filtration Based Technique for Simultaneous SEM and TEM Sample Preparation for the Rapid Detection of Pathogens

**DOI:** 10.3390/v6093458

**Published:** 2014-09-19

**Authors:** Daniel R. Beniac, Christine G. Siemens, Christine J. Wright, Tim F. Booth

**Affiliations:** 1 Viral Diseases Division, National Microbiology Laboratory, Public Health Agency of Canada, Winnipeg, MB R3E 3P6, Canada; E-Mails: christine.siemens@phac-aspc.gc.ca (C.G.S.); christine.j.wright@phac-aspc.gc.ca (C.J.W.); tim.booth@phac-aspc.gc.ca (T.F.B.); 2 Department of Medical Microbiology, University of Manitoba, Winnipeg, MB R3E 0W3, Canada

**Keywords:** scanning electron microscopy (SEM), transmission electron microscopy (TEM), negative staining, filtration, vaccinia

## Abstract

Diagnostic electron microscopy for infectious diseases has the advantage that “everything” in the specimen can be observed, without *a priori* knowledge of the likely identity of the microorganisms present in the sample. The classical specimen preparation method used employs a droplet of sample, which allows particles to adsorb to a support film, and is subsequently negative stained. This “grid on drop” procedure has a sensitivity range of approximately 10^6^ viruses per mL if no enrichment procedures are used. In the current investigation we present a novel use of filtration that allows us to detect viruses at concentrations as low as 10^2^ viruses per mL. We present here methods based on filtration, in which total virus, and not virus concentration, is the limiting factor for detection. We show that filtration is more sensitive than conventional negative staining and can detect as few as 5 × 10^3^ particles per sample.

## 1. Introduction

Electron microscopy was developed in the 1930s to detect objects that could not be resolved by light microscopy [1,2]. Tobacco mosaic virus was one of the first viruses identified in early microscopes [3,4]. Although the quality of the images was poor in comparison to modern instruments, this represented a major advancement in viral diagnostics. The standard virus preparation procedure still used today, known as “negative staining” was developed in the late 1950s and has a sensitivity limit of 10^6^ virus per mL [5]. This sensitivity limit is improved by the use of airfuge ultracentrifugation [6], which when applied to viral samples, such as vaccinia, have established a detection limit of 5 × 10^4^ poxvirus particles per mL [7]. The advantages of electron microscopy are the rapid speed of the process, which takes 10–15 min and the large size range of samples that can be processed. Moreover, both liquid and tissue samples can be analyzed and no prior information is required, as in the case of nucleic acid or antibody based assays [8,9]. In the advent of a disease outbreak or the deliberate release of a pathogenic agent, electron microscopy is one of the rapid front line tests that should be performed as part of the rapid response to these events.

In clinical diagnostic electron microscopy two techniques are most often applied, negative staining for aqueous suspensions, and thin sectioning of cellular and tissue samples [10]. In this investigation we focused on aqueous pathogenic suspensions. We have developed a protocol in which we have coupled negative staining for transmission electron microscopy (TEM) with a filtration-based protocol for scanning electron microscopy (SEM). This hybrid protocol is rapid, and simultaneously prepares SEM and TEM specimens. The protocol can process sample volumes from 10 μL up to 50 mL, and concentrates them on to the SEM filter and TEM grid in as little as one rapid step for subsequent observation.

## 2. Materials and Methods

### 2.1. Growth, Purification and Titration of Modified Vaccinia Ankara Virus

Baby hamster kidney fibroblast cells (BHK-21:ATCC) were grown to 80% confluence in high-glucose Dulbecco’s Modified Eagle’s Medium supplemented with 10% fetal bovine serum (FBS) and 1× Penicillin-Streptomycin. The BHK-21 cells were infected with 1 mL of modified vaccinia Ankara strain (MVA) virus (kindly provided by Dr. Jingxin Cao, National Microbiology Laboratory) and incubated for 48 h at 37 °C with 5% CO_2_. The infected cells underwent 3 freeze-thaw cycles in the presence of the growth media, alternating between −80 °C and room temperature. MVA was collected in the supernatant after removing cells and debris by centrifugation at 3000 × g for 3 min. To calculate the titre, the virus suspension was diluted 1:10 to a final dilution of 10^−7^, 200 µL of each dilution was added to a well of a 6 well plate. An agar overlay was applied to each well and the infection was incubated for 48 h where the visible plaques were counted and the final concentration was calculated. (This MVA strain contains a GFP gene that fluoresces in an UV microscope making it easy to determine the number of plaques for counting).

### 2.2. Growth of Leptospira

*Leptospira biflexa* servoar Patoc (kindly provided by Dr. Robbin Lindsay, National Microbiology Laboratory) was grown in Ellinghausen and McCullough media modified by Johnson and Harris (EMJH) (Royal Tropical Institute, The Netherlands). The inoculated culture was placed at 30 °C for 14 days and then stored at room temperature in low light.

### 2.3. Growth and Purification of Ebola

Zaire Ebola virus (kindly provided by Dr. Steven Jones, National Microbiology Laboratory) was propagated, purified and rendered non-infectious as previously described [11,12].

### 2.4. Growth and Purifiaction of Bacteriophage

The bacteriophage infective against *E. coli* 0157:H7 (kindly provided by Raffiq Ahmed, National Microbiology Laboratory) was isolated from sewage filtered through a 0.45 µm filter followed by growth with selected bacteria in phage broth containing nutrient broth (Sigma-Aldrich, Oakville, ON, Canada) and sodium chloride for 2 h at 35 °C. A total of 10 µL of the propagated phage was dropped onto a lawn of *E. coli* on phage agar composed of nutrient broth, sodium chloride, and Bacto agar (Fisher Scientific, Ottawa, ON, Canada). After overnight growth at 35 °C a plug is removed from a plaque and propagated in *E. coli* for 6 h at 35 °C with shaking. Final purification of the phage was achieved by filtration through a 0.8/0.2 µm filter.

### 2.5. Growth of Herpes Simplex Virus Type I

Vero cells (ATCC) were grown in minimum essential medium (MEM) containing 5% FBS. At 80% confluence the cells were infected with 100 µL Herpes Simplex Virus Type I (HSV-1) (kindly provided by Dr. Stephanie Booth, National Microbiology Laboratory). After 24 h of incubation at 37 °C the HSV-1 infected cells were collected in PBS and lysed by 3 rounds of a rapid freeze-thaw cycle alternating between a dry ice-methanol bath and 37 °C. The cell debris was removed by centrifugation at 2000 × g for 5 min and the supernatant containing the virus was collected.

### 2.6. TEM Sample Preparation

Samples were adsorbed for 1 min to a formvar film on a carbon-coated 200 mesh copper grid. The adsorbed samples were washed 3× in distilled water and negatively contrasted with 2% methylamine tungstate (MT) (Nano-W, Nanoprobes, Yaphank, NY, USA), or 2% Uranyl acetate (EMS, Hatfield, PA, USA). Imaging was performed at 200 kV using a FEI Tecnai 20 transmission electron microscope (FEI Company, Hillsboro, OR, USA). Digital images of the specimens were acquired using an AMT Advantage XR 12 CCD camera (AMT, Danvers, MA, USA).

### 2.7. Gold Coated Sample Preparation for SEM Imaging

**Figure 1:**
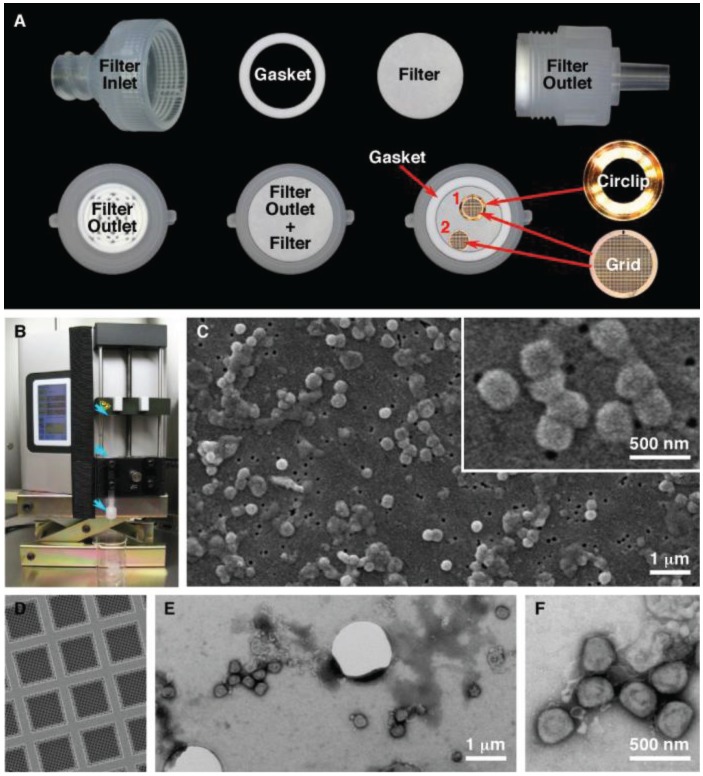
Filtration preparation of vaccinia virus. (**A**) Setup of the filter unit for both SEM and TEM analysis. The quantifoil grids were held in place by the circlip (1) or by the gasket (2); (**B**) Filter unit and syringe (blue arrows) loaded in the syringe pump; (**C**) Characteristic SEM images of vaccinia imaged by SEM; (**D**) Quantifoil support film; (**E**,**F**) Low and high magnification images of vaccinia imaged by TEM.

SPI-pore polycarbonate track etch filters (SPI Supplies, West Chester, PA, USA) with pore sizes of 0.1 μm (leptospira, ebola virus), 0.08 μm (vaccinia virus), and 0.03 μm (HSV-1, bacteriophage) were used as mounting surfaces to view pathogenic organisms in the SEM. The filter membranes were held inside a 13 mm Swinnex® filter unit (Millipore, Billerica, MA, USA) (Figure 1A). Quantifoil R1/4 holey carbon supports (Quantifoil Micro Tools GmbH, Großlöbichau, Germany) on 200 mesh copper grids were further stabilized by carbon evaporation, followed by glow discharge to make the grids hydrophilic (Figure 1). The quantifoil grids were placed on top of the membrane and held by the gasket or in a circlip (Gatan Inc, Pleasanton, CA, USA). The membranes were wetted by pipetting Dulbecco’s phosphate buffered saline (DPBS) into the filter inlet until the chamber was full. To load the sample onto the filter, 100 μL was added to 5 mL DPBS in a 5 mL syringe. After attaching a Luer-Lok™ syringe to the filter holder, the sample was filtered using a Legato 200 syringe pump (KD Scientific, Holliston, MA, USA) at a rate of 1000 μL/min. After filtration, the quantifoil grids were removed, washed with 3 µL and stained for 30 s with MT contrasting stain. The filter units were reassembled and the filters were washed with 2 mL each of 50%, 70%, 85%, 95% and 100% ethanol in increasing concentration. Following the last wash the filter was removed from the filter unit and allowed to air dry for 30 min. The filter was cut in to quarters and placed on a 9 mm carbon disc (SPI Supplies) and mounted on a 3/8” aluminum stub (SPI Supplies). Flash Dry silver paint (SPI Supplies) was used on each of the three corners of the filter to create a contact between the filter and the stub. The samples were removed from the BSC and sputtered with gold using a Quorum Q150R S (Quorum Technologies, East Sussex, UK) containing a 0.1 mm gold target. The chamber was pumped down, purged with argon and sputtered with gold for 120 s while on a rotating stage.

### 2.8. SEM Sample Imaging

All samples were imaged in a JCM-5700 Scanning Electron Microscope (JEOL USA, Peabody, MA, USA). Gold coated specimens were imaged under high vacuum at 6 kV, with an 8 mm working distance and a 30 μm objective lens aperture. Images were collected using the secondary electron detector, the acquisition time per image was 160 s and each image was 2560 × 1920 pixels. The images for the particle counting were collected with an acquisition time of 20 s at 1280 × 960 pixels.

### 2.9. MVA Particle Counts

MVA was serial diluted 10 fold with the series range spanning 5.7 × 10^7^ to 5.7 × 10^4^ infectious particles per mL in PBS. For counting of MVA particles on the TEM a random sampling of 40 grid squares on a 200 mesh formvar grids were scanned and counted by direct visualization. Samples were prepared using the same dilutions for imaging and counting on the SEM. Images were recorded from an equivalent random forty areas of 7786.3 µm^2^ (equivalent to one 200 mesh grid square). In both cases for SEM, and TEM the 40 grid squares (or equivalent in SEM) were collected as follows: 4 unique sample preparations were conducted, and random 10 areas on each (grid or filter) were counted for a total of 40 unique areas counted. The particles were then counted manually, and the results were recorded in Microsoft Excel. For specimens with very high particle counts, the Image J software package was used [13]. To analyze the particles the following software commands were used: Remove Outliers, Smooth, Make Binary, Despeckle, Fill Holes, Dilate, Watershed, and Analyze Particles.

## 3. Results

The initial objective of this technique was to provide a simple rapid method to simultaneously concentrate and mount pathogenic samples for both TEM and SEM analysis. The final protocol that was developed has achieved these goals (Figure 1). This technique permits us to process sample volumes ranging from 10 μL up to 50 mL by filtration. This produces a concentrated sample that has been deposited on both an SEM membrane and a TEM grid. During the development of the procedure we made several modifications to improve the technique. Improvements were obtained by using a syringe pump over manual operation, which gave more consistent filtering. When the procedure is scaled up and used in a laboratory daily, multiple samples (in tens to the hundreds) can be processed. A pump that can process more then one syringe at the same time gives higher throughput, is reproducible, and relies less on operator skill. The initial test results also demonstrated that the syringe/filter unit had to be held in a vertical position, to give an even spreading of the pathogen on the SEM filter that was thus kept in a horizontal position. This also allowed a TEM grid to be placed on the surface of the SEM membrane. In order to hold the grid in position during filtration a gasket or metal circlip was used. This prevents the grid, (which is a thin foil) from moving around within the filter unit. Finally it was essential to “prime” the void volume above the SPI-pore filter once the filter unit is assembled by filling this chamber in the sealed filter unit with an aqueous buffer before the sample is filtered through the filter unit. This reduces turbulence within the filter unit giving an even particle distribution for imaging by microscopy.

**Figure 2:**
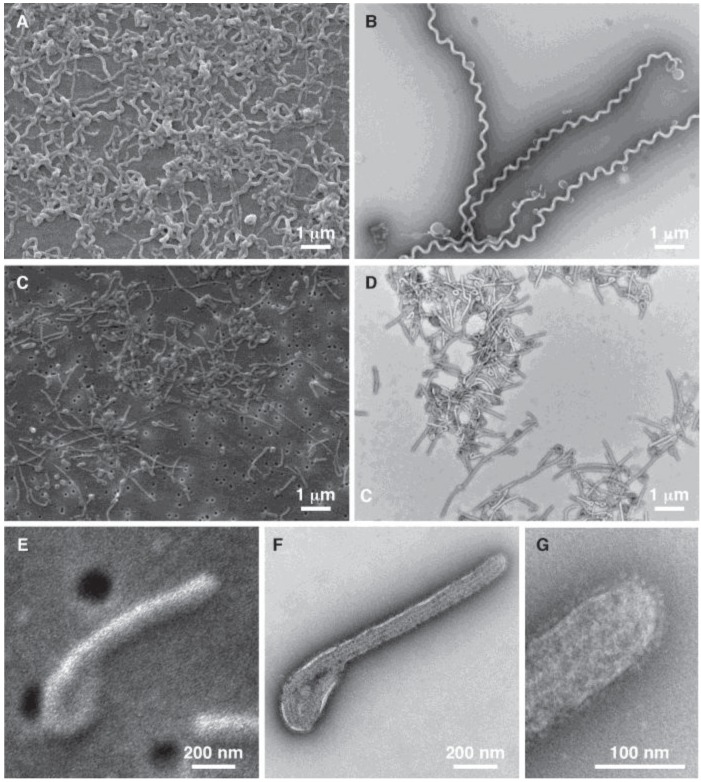
Diagnostic imaging of filamentous pathogens. SEM (**A**,**C**,**E**) and TEM (**B**,**D**,**F**,**G**) images are presented of *Leptospira biflexa* (**A**,**B**); and Ebola virus (**C**–**G**); Images (**A**–**D**) are all presented at the same magnification to illustrate the ability to easily differentiate these two pathogens regardless of the type of electron microscope used to detect the pathogens; Images (**E**–**G**) are presented at higher magnification to show the high resolution structural details these two imaging techniques can identify.

The initial tests were conducted using vaccinia as a model system. Based on this we were able to demonstrate that the system achieved our goal of providing a platform for simultaneous TEM and SEM sample preparation. The next proof of concept for the technique required us to test the versatility of the system to process a variety of pathogens, and to achieve results comparable to the classic “grid on drop” negative staining TEM test. Our first test was to confirm the ability to identify and distinguish between two different samples, one containing an enveloped filamentous virus, and the other a filamentous bacterial sample (Figure 2). This demonstrates the ability to handle both viral and bacterial samples, and to differentiate between two filamentous microbial samples, Ebola virus and *Leptospira biflexa*, based on morphology. The initial assessment of these images shows the ability of both TEM and SEM to identify these specimens regardless of the staining and microscopic observation procedures used. In this case the Ebola virus and *Leptospira biflexa* can be easily differentiated, since the Ebola filamentous viruses are considerably smaller in both length and diameter, and the *Leptospira biflexa* have a distinctive spiral-shaped morphology due to their two internal flagella. In addition, the TEM images are of higher resolution, so features such as the surface glycoprotein spikes on the Ebola virus can only be observed by TEM.

**Figure 3:**
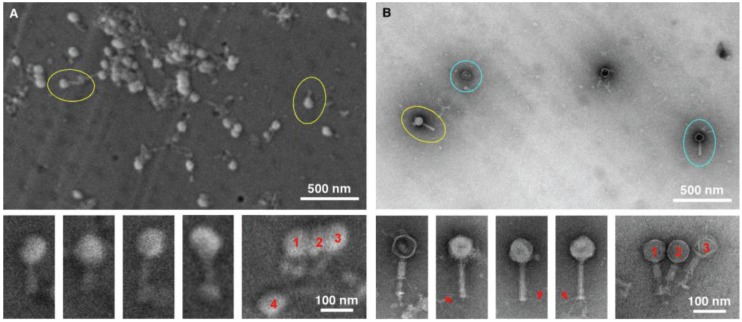
Imaging bacteriophage. SEM (**A**) and TEM (**B**) images clearly demonstrate the ease with which bacteriophage can be detected (yellow circles). Although the TEM images are of superior resolution, for diagnostic purposes the SEM does have the ability to detect the bacteriophage, with the capsid/head and tail clearly visible for both TEM and SEM. However, finer details like the tail fibers (red arrows), and empty heads (blue circles) are only visible by TEM.

**Figure 4:**
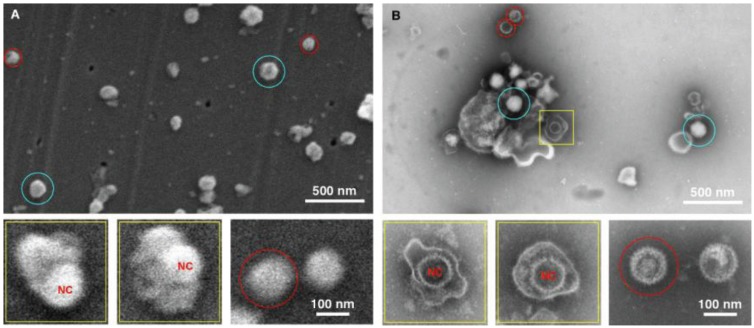
Imaging herpes simplex virus. SEM (**A**) and TEM (**B**) images of the herpes virus are presented, showing intact virus (blue circle), partially disrupted (yellow squares), and nucleocapsids (red circles). In this case both methods detected the virus, however the staining in the TEM images provide a superior diagnostic identification, for example the nucleocapsid (NC) has superior staining in the TEM sample preparation.

The next two tests performed were conducted to specifically compare negative staining and TEM with gold sputtering SEM using two distinctive viral samples, in this case a bacteriophage (Figure 3) and herpes virus (Figure 4). The purpose was to test the ability to detect and distinguish between enveloped and non-enveloped viral particles based on morphology. Both samples were prepared simultaneously, and were of the same particle concentration and volume. In both cases, considerably more viral particles were observed in the SEM-filtered samples, than in the TEM “grid on drop” negative stained samples. We anticipated this, since the sample in the fluid column is concentrated and deposited on the SPI-pore filter in the SEM preparation, whereas only those viral samples adjacent to the formvar film can adsorb to the film in the “grid on drop” sample preparations. As for morphological analysis, the bacteriophage can be identified by both imaging procedures, and the capsid (head) and tail are also clearly identified by both methods. However, the TEM negative staining process has the benefit that stain penetration allows one to differentiate between capsids that are full and empty, and in addition the tail fibers can also clearly be seen in the negative stained samples.

The data for the herpes preparation shown in Figure 4 indicated that the TEM clearly identified the viral sample. This was due principally to the ability of the negative stain to penetrate the viral envelope, and stain the viral nucleocapsid in the interior of the virus. Nucleocapsids which were not inside the virus were also clearly identifiable, and the individual capsomers can be identified. As for the SEM data only surface morphology is evident, the images are therefore suggestive in nature with regards to the interior contents. For example there is a distinctive bulge in some of the intact viruses that is the size of the nucleocapsid. This makes an SEM-only based diagnosis difficult for this enveloped virus sample, and this demonstrates the utility of this technique which integrates both TEM and SEM preparation in the procedure.

**Figure 5:**
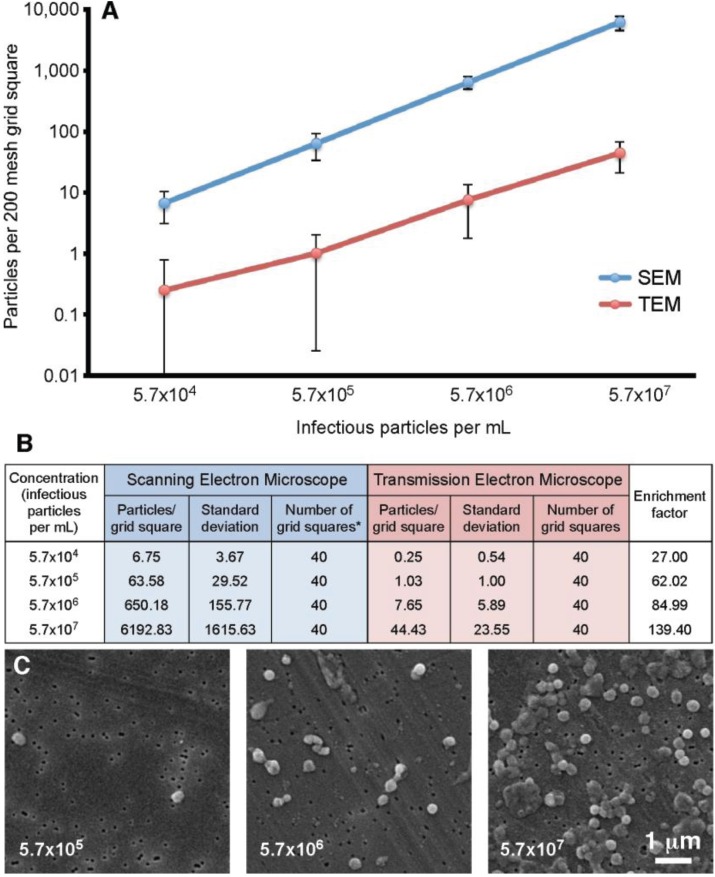
Particle number on a 200 mesh grid square prepared using the “grid on drop” TEM method compared to the SEM filtration protocol. (**A**) Standard TEM sample adsorption (red) is compared to the SEM-filtration method (blue); (**B**) The particle counts that correspond to the graph in (**A**) are presented here along with the enrichment factor obtained by using the filtration procedure; * For the SEM data 63 images were recorded in an area equivalent to that of a 200 mesh grid square; (**C**) Representative SEM images of vaccinia recorded at various concentrations.

Our previous experiments demonstrated that the filtration method appeared to give an increased particle count compared to the standard “grid on drop” negative staining procedure. In order to quantitate this observation, a vaccinia virus dilution series was analyzed. The concentrations ranged from 5.7 × 10^4^ to 5.7 × 10^7^ infectious particles per mL. For this test four 200 mesh TEM grids were prepared and ten random grid squares per grid were selected. For the SEM component, four filters were prepared, and on each ten random areas of 7786.3 µm^2^ were counted (63 adjacent images recorded at 10,000× is equivalent to the area of a 200 mesh grid square). The results of this analysis are presented in Figure 5. This data demonstrated that the filtration protocol has an enrichment factor that ranges from 27.0 to 139.4 times that of the “grid on drop” method. In general the enrichment shows approximately a 100-fold increase in particle count with the SEM filtration technique compared to the TEM “grid on drop” negative staining technique.

**Figure 6:**
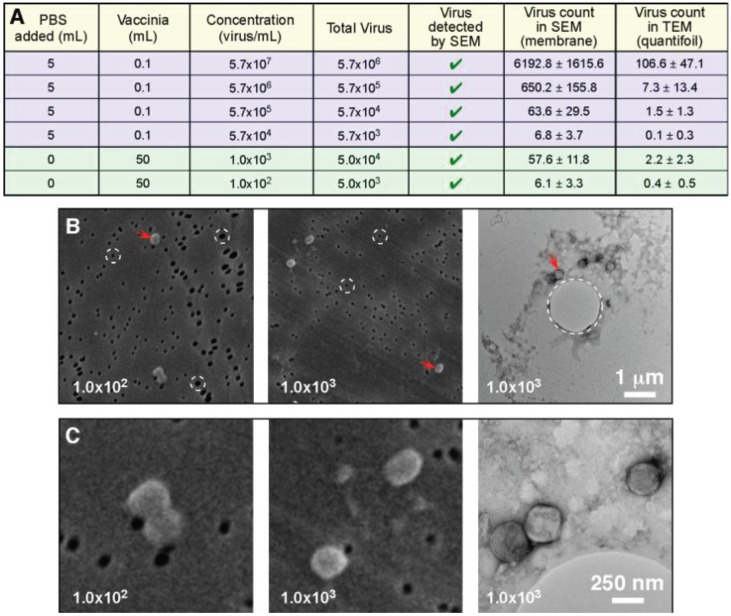
Total particle count and not concentration determine the ability to detect a pathogen. (**A**) The data from Figure 5 (blue: 0.1 mL sample volume) has been presented to include the total virus present in the samples; This data has been supplemented by two additional samples that were extremely low concentration, but were considerably larger sample volumes (green: 50 mL sample volume); Low magnification (**B**), and higher magnification images (**C**) clearly demonstrate the ability to detect the low concentration pathogens by both TEM and SEM; In (**B**) we have high-lighted the pores in both of the support filters/films (circles with white dashed lines). The SPI-pore filter pores are ~80 nm and do not permit vaccinia to pass through, whereas the ~1 µm pore in the quantifoil support film can permit passage of the vaccinia.

The next test conducted in this investigation looked at the ability of the filtration protocol to process large volume samples (50 mL), which were at extremely low concentrations (1.0 × 10^3^ infectious particles per mL range, and 1.0 × 10^2^ infectious particles per mL range). In this experiment both the TEM and SEM samples were filter preparations: We were comparing the ability to detect the virus, and the time taken. For the 1.0 × 10^3^ infectious particles per mL range sample it took approximately 1 min to positively identify vaccinia by SEM, and 15 min to identify it by TEM. With the lower concentration 1.0 × 10^2^ infectious particles per mL range sample it took approximately 5 min to positively identify vaccinia by SEM, and 45 min were required to identify it by TEM (our standard cut off time for identification is 30 min). Since the SEM positive falls well within the test time limit, and the TEM identification occurred just outside of our test time limit, we consider this to be the practical detection limit for this procedure. This indicates that the SEM filter has a higher retention of viral particles than the quantifoil grid. We suspect that the larger 1 µm holes in the quantifoil allow vaccinia (which is smaller, ~360 × 270 × 250 nm) to pass through, whereas vaccinia cannot pass through the 80 nm pore of the SPI-pore filter. It should also be noted that compared with the previous experiment where we worked with vaccinia in the 5.7 × 10^4^ to 5.7 × 10^7^ infectious particles per mL range, the increased sample volume compensates for the low concentration. Therefore, the “total virus” used in these experiments is the actual limiting factor for detection, and not the concentration. Figure 6 shows that the total virus is in the same range for both the high and low concentration samples.

The final test in this investigation looked at the ability of the filtration protocol to detect small volume samples. In this test a 10 µL sample volume of vaccinia was used. The resulting SEM particle counts for this test were as follows: 5.7 × 10^7^ virus/mL = 539.8 ± 298.3 particles/grid square, 5.7 × 10^6^ virus/mL = 79.4 ± 34.8 particles/grid square, 5.7 × 10^5^ virus/mL = 8.2 ± 3.3 particles/grid square, 5.7 × 10^4^ virus/mL = 0.6 ± 0.9 particles/grid square. These results demonstrated the ability of this filtration protocol to process a wide range of samples ranging from 50 mL down to 10 µL sample sizes. In this experiment the 10 µL sample size was processed by injecting the 10 µL virus sample directly into 5 mL of PBS and then this was loaded into a 5 mL syringe and processes exactly the same as the other samples.

## 4. Discussion

In this investigation we have demonstrated the utility of a filtration-based protocol for simultaneous preparation of TEM and SEM samples. SEM can be used to investigate pathogens [14], however the majority of electron microscopy based diagnostics have historically relied on TEM. We have developed a hybrid technique which gives us the best of both methods. When compared to other diagnostic techniques the negative stain TEM technique is the fastest taking approximately 15 min [15]. This is in contrast to nucleic acid amplification methods (1–4 h), cell culture detection (2–14 days), immune electron microscopy (2–4 h), ELISA (2–3 h), and immune light microscopy (2–3 h) [15]. Taking this into consideration we designed our filtration-based protocol with speed in mind. Our procedure takes 10 min to produce a TEM grid ready for imaging, and 25 min to produce the SEM filter ready for imaging. Then an additional 5–30 min of imaging time is usually required to identify a pathogen. When dealing with a diagnostic emergency, due to an unusual disease outbreak, or the intentional release of a pathogenic agent, rapid diagnostic protocols are essential as part of the front line response. The speed of this new technique meets this requirement.

For most negative stain based TEM sample preparative methods, a sample volume of 5–100 µL is applied to a grid. For the “grid on drop” method a sample volume of 5–50 µL is used, and for the airfuge method approximately 100 µL of sample is required. These are the minimum volumes that these two procedures require. The filtration method that we have developed can process essentially any volume, and the pathogen being investigated will be concentrated on the surface of the SEM filter and TEM grid. Due to this large volume range, this procedure could be applied to fluid samples such as urine, which can be low pathogen concentration/large volume samples. In the case of these larger volumes a concern to be addressed is the presence particulate materials in the specimen that could block or overload the pores in the membrane. This difficulty could be improved by applying a low speed clearing spin prior to filtration. An alternative would be filtration of the sample two times, once with larger pore sizes to remove the large particulates, and then with smaller pore sizes that would capture the virus. In this investigation we used virus specimens in PBS to test the viability of the procedure. Future work will involve the processing of common fluids such as blood, urine, and vesicular fluid. In general this method is not limited by concentration due to the wide range of volumes processed, rather we are limited by total pathogen present. The results in Figure 6 demonstrated that we could count virus particles in the low concentration 50 mL, 10^2^ virus/mL sample that contained a total of 5 × 10^3^ virus particles. Similar counts were obtained for the 100 µL sample with 5.7 × 10^4^ virus/mL that contained 5.7 × 10^3^ total virus particles. When detection of virus in the low concentration 10^2^ virus/mL sample was tested with a cut off time for detection of 30 min per grid the virus was detected by SEM in this time frame, and the TEM sample was detected just outside of the time frame. Therefore, we consider 5 × 10^3^ total virus particles per test to be the current detection limit for this technique.

We also tested the ability of filtration to analyze small sample volumes. To do this we processed and imaged vaccinia samples by SEM that was only 10 μL. The concentration range tested was: 5.7 × 10^7^, 5.7 × 10^6^, 5.7 × 10^5^, and 5.7 × 10^4^ virus/mL. The particle counts associated with these were 539.8 ± 298.3, 79.4 ± 34.8, 8.2 ± 3.3, and 0.6 ± 0.9 particles/grid square, respectively. Interestingly if one accounts for the 10 µL sample volume and concentration the total virus loaded in the above tests would correspond to 5.7 × 10^5^, 5.7 × 10^4^, 5.7 × 10^3^, and 5.7 × 10^2^ total virus, respectively for these four samples. If we then compare across the three sample volumes (50 mL, 100 µL, and 10 µL) we get very similar results. For example: 50 mL (5.0 × 10^4^ total virus) had 57.6 ± 11.8 particles/grid square, 100 µL (5.7 × 10^4^ total virus) had 63.6 ± 29.5 particles/grid square, 10 µL (5.7 × 10^4^ total virus) had 79.4 ± 34.8 particles/grid square. Similarly: 50 mL (5.0 × 10^3^ total virus) had 6.1 ± 3.3 particles/grid square, 100 µL (5.7 × 10^3^ total virus) had 6.8 ± 3.7 particles/grid square, 10 µL (5.7 × 10^3^ total virus) had 8.2 ± 3.2 particles/grid square. These results demonstrate the consistency and scalability of sample volume that this technique can accommodate.

One variation that we observed in this investigation was that more viral particles were retained on the SPI-pore filter then on the quantifoil TEM grid. When TEM and SEM samples were prepared simultaneously, the reduction in particle count could be measured, as seen in the Figure 6. We interpreted this to be caused by the pore size. The SPI-pore “track etch” filters are fabricated with pores that range from 10 nm to 20 µm in diameter, whereas the quantifoil grids we used have pores ranging from 0.6 to 5 µm in diameter. Vaccinia can easily pass through the 1 µm holes in the quantifoil we used, making it difficult to ascertain the reason for the lower vaccinia concentration on the grid. Was this an issue of the virus particles not sticking to the grid for some reason, or did they just pass through the holes in the quantifoil? We tried using grids with solid formvar support film, but these tended to have poor staining and even less vaccinia was counted than on the quantifoil. We interpreted this to be caused by a lack of fluid flowing past the solid film in the regions adjacent to the TEM grid (*i.e.*, the pores are needed to let the liquid to flow by, but they must be small enough to catch the virus). We also compared using a grid held by the gasket of the filter unit, or in a circlip (Figure 1). The thought was that the circlip which is “cup” shaped and holds the grid would function like a funnel during filtration to help increase the number of virus particles on the quantifoil. At present using the circlip or gasket made no difference to the virus density observed on the TEM grid. This again leads us to postulate that large holes in the support film permit the virus to merely pass through and not be retained by the filter. This virus loss we have observed needs to be further studied in order to increase virus retention on the quantifoil TEM grid. There are two ways to approach this. Firstly, to test the recently fabricated TEM grids with a smaller 600 nm pore size to test for viral particle retention. Secondly, to test the effect of liquid flow rate on virus particle retention. In the current investigation this was kept constant at 1000 µL/min in the syringe in order to generate a baseline for the comparison of the detection sensitivity of various biological samples with SEM and TEM. In future, a slower flow rate may improve virus particle retention.

A recent study by Laue and Bannert [7] compared standard negative stain preparations to those enriched by airfuge centrifugation for vaccinia and *B. subtilis* spore detection by TEM. The airfuge technique essentially spins the majority of the sample down onto the EM grid for observation. Their vaccinia results cannot be directly compared to ours, since different grids were used (400 mesh instead of 200 mesh) as well as different concentrations and sample volumes. Nevertheless it is interesting that Laue and Bannert achieved airfuge enrichments of 113 times for the 10^6^ particles per mL, and 34 times for the 10^5^ particles per mL specimens, compared to standard negative staining. In our study using filtration we achieved enrichments of: 139.40 times for the 5.7 × 10^7^ samples, 84.99 times for the 5.7 × 10^6^ samples, 62.02 times for the 5.7 × 10^5^ samples, and 27 times for the 5.7 × 10^4^ infectious particles per mL samples when compared to standard negative staining (Figure 5). We found a similar trend to Laue and Bannert with the lower concentration samples having a lower enrichment value. It should be noted that the differences in enrichment factor *versus* particle concentration that we have detected are primarily due to the large standard deviation in the data. If one looks at the lines in Figure 5A (blue and red) the last three data points/lines are fairly parallel. The first point appears a bit off this parallel line. The reason for this is that the vaccinia virus tends to aggregate and one can have areas with 5–10 viruses all together in a clump of virus. This subsequently will count as 5–10 virus particles which will affect the data analysis. In this experiment, for example for the highest concentration TEM data would have (44.43 viruses × 40 grid squares = 1760 total viruses). If one adds a five virus aggregate this would give1765 viruses which would not be a major influence on the data. However if we then apply the same to the low concentration TEM data then we would have (0.25 viruses × 40 grid squares = 10 total viruses). If one adds a five-virus aggregate this would give 15 viruses, which would influence data by a factor of 1.5 times. This shows that the lower concentration TEM sample is sensitive to the aggregates of the virus that we detect, resulting in an elevated mean value, and larger standard deviation. This affects the data since the particle distributions are not homogeneous due to this aggregation.

Similarly Biel and Gelderblom [15] reported that airfuge centrifugation produced and enrichment over negative staining in the range of 10–100 times. Both airfuge centrifugation and filtration essentially concentrate the sample down onto the grid, whereas “grid on drop” methods rely on the adsorption/diffusion of samples onto the support film. In both cases (airfuge and filtration) the enrichment protocols achieved similar enrichments of 30 to 140 times over the concentration ranges that were tested. In addition, many particles have physical properties that can sometimes repel them from support films, or cause them to aggregate, making detection and counting difficult when using adsorption methods. Particle adsorption characteristics can often be improved by glow discharge of the support films before use. However, centrifugation and filtration do not rely on this irregular process that is difficult to standardize. Thus filtration can give more consistent, reproducible results than adsorption, allowing improved sensitivity and more accurate quantitation than centrifugation for diagnostic electron microscopy.

## 5. Conclusions

Electron microscopy sample preparation is relatively quick, and a wide range of pathogens both viral and bacterial can be easily identified using these techniques. This coupled with the fact that electron microscopy dose not require any prior knowledge of a sample (*i.e.*, no primers, antibodies, required to process a sample) makes the technique an essential part of the unknown pathogen detection process. In this investigation we have presented the results of a dual SEM-TEM filtration protocol, which has the advantage of being fast, and the ability to detect low concentration pathogens. This technique can be used to augment the standard “grid on drop” negative staining protocol used in most diagnostic electron microscopy laboratories.
